# The role of artificial intelligence in the endoscopic diagnosis of esophageal cancer: a systematic review and meta-analysis

**DOI:** 10.1093/dote/doad048

**Published:** 2023-07-21

**Authors:** Nadia Guidozzi, Nainika Menon, Swathikan Chidambaram, Sheraz Rehan Markar

**Affiliations:** Department of General Surgery, University of Witwatersrand, Johannesburg, South Africa; Department of General Surgery, Oxford University Hospitals, Oxford, UK; Academic Surgical Unit, Department of Surgery and Cancer, Imperial College London, St Mary’s Hospital, London, UK; Department of General Surgery, Oxford University Hospitals, Oxford, UK; Nuffield Department of Surgery, University of Oxford, Oxford, UK

**Keywords:** cancer screening, endoscopic imaging, esophageal cancers, robotics

## Abstract

Early detection of esophageal cancer is limited by accurate endoscopic diagnosis of subtle macroscopic lesions. Endoscopic interpretation is subject to expertise, diagnostic skill, and thus human error. Artificial intelligence (AI) in endoscopy is increasingly bridging this gap. This systematic review and meta-analysis consolidate the evidence on the use of AI in the endoscopic diagnosis of esophageal cancer. The systematic review was carried out using Pubmed, MEDLINE and Ovid EMBASE databases and articles on the role of AI in the endoscopic diagnosis of esophageal cancer management were included. A meta-analysis was also performed. Fourteen studies (1590 patients) assessed the use of AI in endoscopic diagnosis of esophageal squamous cell carcinoma—the pooled sensitivity and specificity were 91.2% (84.3–95.2%) and 80% (64.3–89.9%). Nine studies (478 patients) assessed AI capabilities of diagnosing esophageal adenocarcinoma with the pooled sensitivity and specificity of 93.1% (86.8–96.4) and 86.9% (81.7–90.7). The remaining studies formed the qualitative summary. AI technology, as an adjunct to endoscopy, can assist in accurate, early detection of esophageal malignancy. It has shown superior results to endoscopists alone in identifying early cancer and assessing depth of tumor invasion, with the added benefit of not requiring a specialized skill set. Despite promising results, the application in real-time endoscopy is limited, and further multicenter trials are required to accurately assess its use in routine practice.

## INTRODUCTION

Esophageal cancer is the eighth leading cause of cancer death worldwide, with a notoriously insidious onset, aggressive tumor biology and a reported 5-year survival of ~15%.^1^^,^^2^ The depth of invasion of esophageal mucosa correlates with possible lymph node involvement, thereby stratifying treatment options. Curative endoscopic resection is offered when tumor invasion is limited to the most superficial third of the submucosa (SM1), whereas, deeper invasion beyond this (SM2) requires chemoradiotherapy or surgical resection or a combination of therapies.^3^^,^^4^ Early diagnosis of esophageal cancer is often limited by a lack of symptoms, but also by subtle macroscopic appearances at endoscopy, often requiring skilled endoscopists to identify and biopsy areas of suspicion.^5^

Artificial intelligence (AI) refers to machine intelligence. Deep learning and machine learning are important components of AI. Deep learning comprises layers of features that are learned from data using a general-purpose learning procedure.^6^^,^^7^ Machine learning refers to a system that can be taught to discriminate characteristics of data samples and then use this information to interpret new information.^6^^,^^7^ Convolutional neural networks (CNNs) are supervised machine learning models made up of multiple network layers that function by extracting key features from an input and provide final classification through connected layers as an output.^8^ Machine learning with support vector machines is based on researchers manually identifying features of interest as input data, in order to train a system to recognize discriminative features, and then produce appropriate outputs.^8^ These AI algorithms have been used in multiple studies to assist with the computer-aided diagnosis (CAD) of esophageal cancer.

Given the wide range of AI modalities, the variability in expertise in endoscopy and the diagnostic subtleties of esophageal malignancies, there is an increasing clinical need for AI to support the endoscopic diagnosis of esophageal cancer. In some cases, AI has been shown to be superior to inexperienced endoscopists in diagnostic capabilities, with the additional benefit of reducing inter-observer variability and minimizing human error.^6^^,^^8^ This article aims to provide an up-to-date summary of the available published evidence, in the form of a meta-analysis and systematic review of the current literature using AI in endoscopic diagnosis of esophageal cancer.

## METHODS

### Literature search

The search methodology was defined according to the Preferred Reporting Items for Systematic Reviews and Meta-Analyses (PRISMA) guidelines.^9^ A systematic literature search was carried out using Pubmed, MEDLINE and Ovid EMBASE databases (date range: 1992 to 6 January 2023) using the following search strategies with standard Boolean operators: ‘artificial intelligence,’ ‘endoscopy,’ ‘machine learning,’ ‘esophageal cancer’ and ‘oesophageal cancer.’ Furthermore, the reference lists of included articles and review articles were searched for additional studies. Only English language studies that used either still endoscopic images or videos were included in the study. Some studies using AI to examine histological slides were excluded, as well as studies reporting on the use of AI-assisted endoscopic sponge cytology.

### Statistical analysis

All statistical analyses were performed using STATA/SE, version 16.0 (StataCorp LLC, College Station, TX). The overall pooled estimate of sensitivity and specificity with their corresponding 95% confidence interval (95% CI) was calculated using the random-effects model by the metandi command in STATA/SE. Sensitivity was defined as the proportion of patients with esophageal cancer that were correctly confirmed by AI, while specificity was defined as correctly identifying patients without the disease. Forest plots were used to visualize the variation of the diagnostic parameters effect size estimates with 95% CI and weights from the included studies.

## RESULTS

**Fig. 1:**
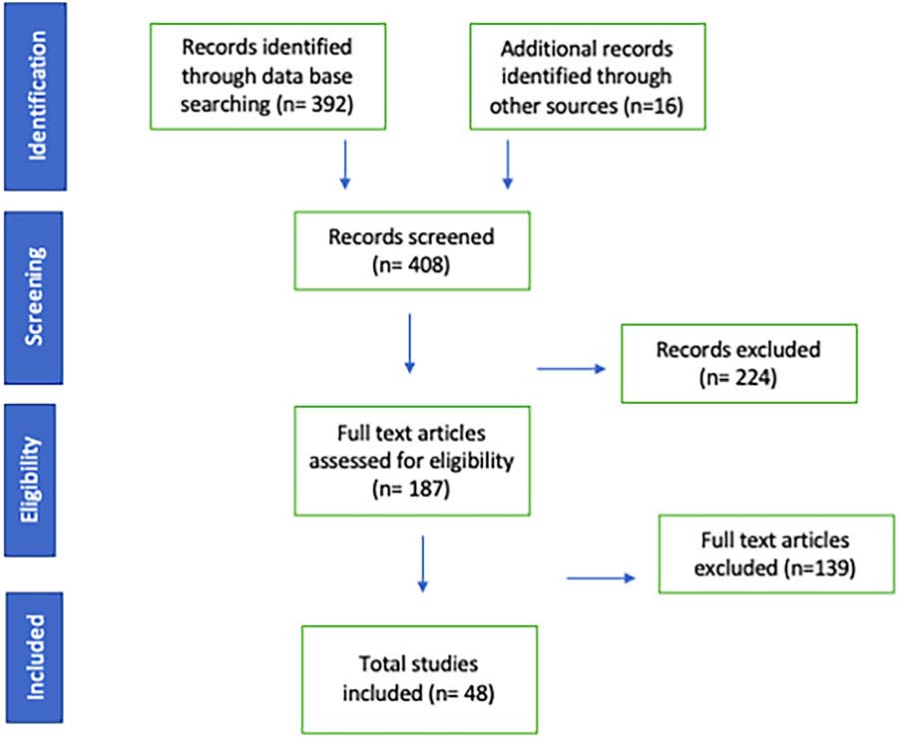
PRISMA diagram showing the sequence of article screening and selection.

Figure 1 illustrates the search methodology used in this study. In total, 48 articles were included in the qualitative and quantitative analysis of AI use in endoscopy. The meta-analysis is separated into AI algorithm application in the diagnosis of esophageal squamous cell carcinoma (ESCC) and esophageal adenocarcinoma (EAC).

### Esophageal squamous cell carcinoma

#### Diagnosis

**Fig. 2:**
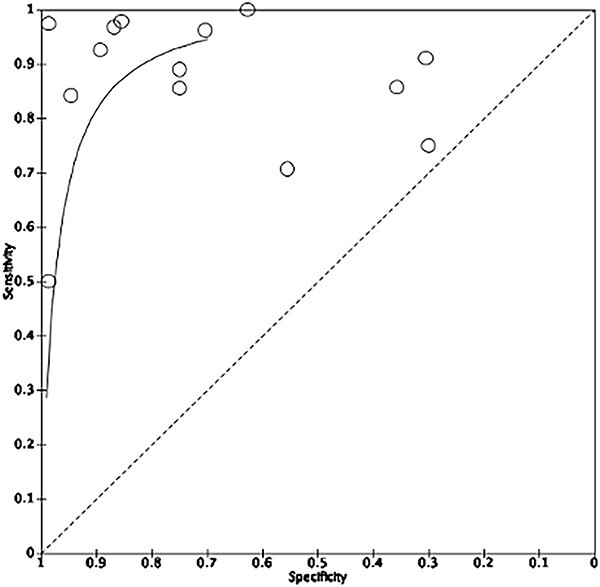
Summary of AI use in endoscopic diagnosis of squamous cell carcinoma.

**Fig. 3:**
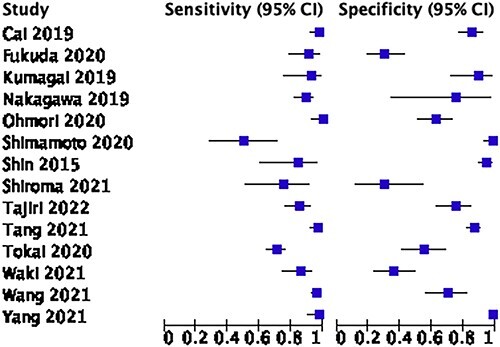
Forest plot showing the results of AI use in endoscopic diagnosis of squamous cell carcinoma.

In total, 24 studies explored the use of AI in the endoscopic diagnosis of ESCC. Fourteen retrospective studies had sufficient data to be included in this meta-analysis. A total of 1590 patients were used in the validation cohort of the studies. The pooled sensitivity and specificity were 91.2% (84.3–95.2%) and 80% (64.3–89.9%) (Figs 2 and 3). The meta-analysis included six studies using AI algorithms in still images,^3^^,^^10–14^ three studies using video images^15–17^ and five studies using a combination of still images and videos.^18–22^ The benefit of using videos in the assessment of AI technology is the realistic application of the system for diagnostic purposes.

The majority of the literature used CNN AI systems in the assessment of early ESCC. Twelve out of fourteen studies used either white-light imaging (WLI) or narrow-band imaging (NBI) images or a combination of both^3^^,^^11^^,^^12^^,^^14–22^ with four studies specifically comparing magnifying endoscopy (ME) and non-ME images.^12^^,^^14^^,^^19^^,^^20^ Wang *et al*.^15^ used a single shot multibox detector (SSD) model in WLI and NBI for esophageal neoplasms, SSD showed higher specificity in diagnosing WLI images but higher sensitivities in NBI images with similar accuracies. Tang *et al*.^21^ developed a system using WLI exclusively, the benefit lying in the convenience and easy availability of this type of imaging, as opposed to using NBI. Their results showed accuracy of 91.3%, sensitivity of 97.9% and specificity of 88.6%, these statistical values outperformed the endoscopists.

In order to assess the capabilities of the AI system, 12 studies compared AI diagnostic performance with a variety of skilled endoscopists.^3^^,^^11^^,^^13^^,^^14^^,^^16–22^ Eight of these studies showed that AI technology was superior to endoscopists in the diagnosis of ESCC.^3^^,^^11^^,^^16–19^^,^^21^^,^^22^ Five further studies analyzed the efficacy of combining AI technology assist to endoscopy and evaluated whether it improved endoscopists accuracy, sensitivity and specificity.^11^^,^^16^^,^^17^^,^^20^^,^^21^ Yang *et al*. showed that using AI assistance improved the diagnostic accuracy of novice endoscopists to comparable levels to that of experts (85.7%), while Cai *et al*. showed that even senior endoscopists could improve their accuracy rates from 88.8 to 93.5% when AI technology was used as an adjunct to endoscopy.^11^^,^^20^

#### Depth of invasion

Three out of the fourteen studies were retrospective reviews focusing on using AI algorithms to identify depth of tumor invasion in ESCC.^3^^,^^12^^,^^19^ Traditional options for detecting invasion depths include WLI, magnifying endoscopy with NBI (ME-NBI) and endoscopic ultrasound (EUS). These modalities have accuracies of 71.4 and 65.3% for WLI and ME-NBI, respectively, while EUS has sensitivity of 85% and specificity of 87% for T1a tumors.^3^ Nakagawa *et al*. and Tokai *et al*. both used deep learning CNN systems to assess invasion depth of tumors <200 μm and >200 μm (SM1 and SM2).^3^^,^^12^ Nakagawa *et al*.^12^ validated their system on non-magnified images (WLI/NBI), magnified images and then iodine-stained images in 155 patients with histologically proven ESCC. Their system was then compared with endoscopists’ capabilities using the same dataset.^12^ The AI system diagnosed all the images in 29 seconds compared with 115 minutes required by the endoscopists. The diagnostic performance of the AI system was comparable with experienced endoscopists.^12^ Tokai *et al*.^3^ developed a system that took an even shorter period of time (10 seconds) to diagnose 291 NBI and WLI images. The features used to indicate deeper invasion in this study included thickness, marginal elevation, red color and apparent lesion depression.^3^ AI NBI image detection was more sensitive than WLI, with overall accuracy of 80.9% in this study.^3^ Shimamoto *et al*.^19^ also used videos of WLI and blue laser images (similar to NBI) in their study. They analyzed non-ME tumor protrusions, depression and hardness of lesions, followed by ME evaluation of superficial vascular architecture and then iodine staining to delineate cancer spread.^19^ This study showed higher accuracy and sensitivity in ME diagnosis compared with endoscopists, but lower specificity.^19^ Overall, the AI system showed better performance with ME videos compared with non-ME images.^19^ All of these studies showed promise in accurate detection of tumor invasion, without requiring specific endoscopic expertise.

**Fig. 4:**
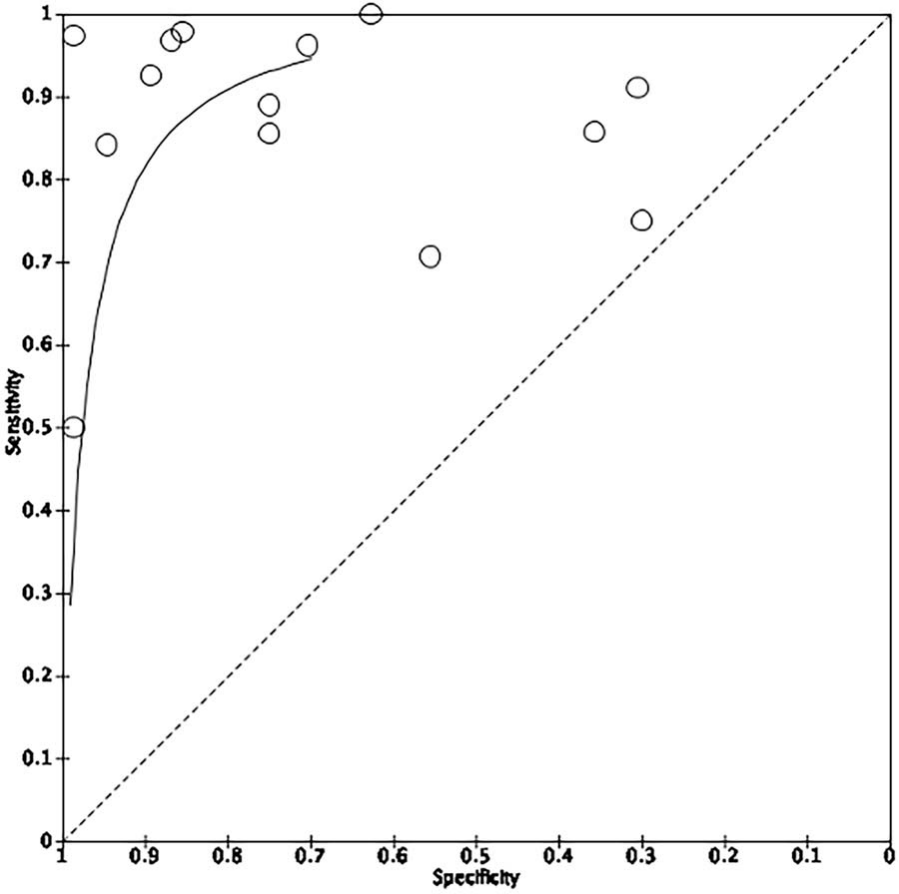
Summary ROC curve showing the results of AI use in endoscopic diagnosis of adenocarcinoma.

### Intrapapillary capillary loops

Intrapapillary capillary loops (IPCL) is an endoscopic microvascular pattern found in atypical esophageal squamous epithelium.^7^^,^^23^ The Japan Esophageal Society (2012) classified IPCL into Types A and B, with Type B vessels further being separated into three classes based on vessel tortuosity and diameter.^23^ IPCL is commonly identified using chromoendoscopy or advanced endoscopic imaging with NBI.^24^ The precise classification in real-time is subjective, inconsistent and reliant on experienced endoscopists. With the increasing volume of work in endoscopy, AI models have been developed to improve the diagnostic accuracy of IPCLs and possibly mitigate the need for expert endoscopists.^7^^,^^23^

Uema *et al*.^23^ developed a CNN system that had accuracy of 84.2% in diagnosing Type B microvascular patterns compared with the average endoscopist’s accuracy of 77.8%. Other studies with promising results in this field include Zhao *et al*.^25^ who used NBI-ME images and AI to discriminate between Type A and Types B1 and B2 patterns, which achieved a classification rate of 87% and mean accuracy of 89.2%. Everson *et al*. used a CNN model, which identified vessel patterns with sensitivity and specificity of 89% and 98%, respectively.^23^^,^^24^ García-Peraza-Herrera *et al*.^26^ also created a detection system for IPCL identification, but their proposed system ResNet-18-CAM-DS had lower accuracy (91.7%) compared with 12 expert endoscopists (94.7%). In 2022, Yuan *et al*.^27^ conducted a multicenter study using ME-NBI images to identify Type A and B1–3 vessels. Their AI system showed combined accuracy of 91.4% for diagnosing IPCL subtypes compared with senior and junior endoscopist’s accuracy of 87.1 and 78.2%, respectively.^27^ AI-assisted endoscopy was shown to reduce diagnostic times for both junior and senior endoscopists, improve inter-observer agreement and improve overall accuracy.^27^ Therefore, the value in AI technology assisting in identifying IPCL patterns lies in the ability to diagnose early ESCC, predict level of invasion, objectively identify pathology and potentially prevent allergic reactions associated with iodine staining in traditional diagnostic methods.^24^

### Esophageal adenocarcinoma

#### Diagnosis

**Fig. 5:**
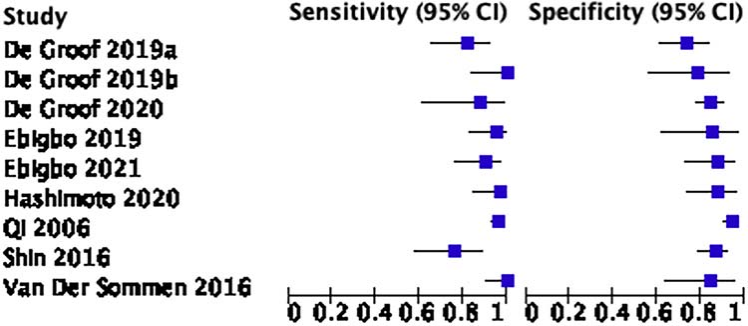
Forest plot of AI use in the diagnosis of adenocarcinoma.

Nine out of fifteen studies including 478 patients had enough statistical information to be included in the meta-analysis of AI to endoscopically identify EAC. The pooled sensitivity and specificity were 93.1% (86.8–96.4) and 86.9% (81.7–90.7) (Figs 4 and 5). Of the nine studies used, five based their system on WLI and two used both WLI and NBI.^28–32^ De Groof *et al*. 2019 was a prospective study, all the other studies were retrospective in nature. The majority of the studies focused on identifying early EAC on a background of Barrett’s esophagus (BE).

The use of AI technology in identifying EAC compared with a diverse range of skill sets of endoscopists was analyzed in six studies.^28–33^ The AI system by van Der Sommen *et al*. was found to be inferior to endoscopists, while in a paper from 2020 Ebigbo *et al*. had comparable outcomes to endoscopists. In 2019 and 2020, de Groof *et al*. found that their AI system was superior to non-expert endoscopists, and in another study by Ebigbo *et al*. and Qi *et al*., they showed overall superior results when compared with endoscopists.^28–33^

### BE surveillance and the diagnosis of early EAC

The endoscopic surveillance of BE is a time- and resource-consuming process due to the need for repeated endoscopies and multi-level biopsies. AI is a crucial adjunct to circumvent these challenges. In 2016, van der Sommen *et al*.^28^ used a machine learning algorithm trained to use color and texture to identify neoplastic lesions in BE. This model had sensitivity and specificity of 83% per image.^28^ Horie *et al*. used a CNN model in eight EAC patients with accuracy of 90% and per case sensitivity of 88% for WLI and NBI and an image processing speed of 0.02 seconds.^34^ In 2022, Knabe *et al*.^35^ used AI to assess the tumor (T) staging of adenocarcinoma in BE in 1020 images. They were able to identify mucosal cancer with accuracy of 68% and larger T3/T4 lesions with accuracy of 73%.^35^

Ebigbo *et al*. used a deep learning system to differentiate between T1a and T1b tumors in still WLE images. The results of the AI system showed superior accuracy (71 vs 70%) and sensitivity (77 vs 63%), however a lower specificity (64 vs 78%).^36^ Ebigbo *et al*.^32^ also used a CAD ResNet model utilizing two databases to improve identification of BE endoscopically. The CAD model utilized still images and achieved WLI and NBI sensitivities and specificities of 97% and 88% and 94% and 80%, respectively, in one database. In the second database, sensitivities and specificities for WLIs were 92% and 100%.^32^ The results from dataset 1 outperformed 11 out of 13 endoscopists.^32^ Ebigbo *et al*. then went on to use their AI system in real-time endoscopy. The AI system showed results comparable to experienced endoscopists with accuracy of 89.9%, sensitivity of 100% and specificity of 83.7%.^31^ Mendel *et al*. used one of the same databases as Ebigbo *et al*. consisting of 39 patients and achieved sensitivities and specificities of 94% and 88% with their AI technology for WLI.^37^

Ghatwary *et al*.^38^ used CNNs to detect abnormal areas of the esophagus in 100 high-definition white-light endoscopy (HD-WLE) images. Four methods for feature extraction were used—regional-based CNN (R-CNN), Fast R-CNN, Faster R-CNN and SDD. The SDD was the most successful method, achieving sensitivity of 96% and specificity of 92%.^38^ The SDD also predicted cancerous images in 0.1–0.2 seconds, thereby being the fastest method.^38^

In 2019, de Groof *et al*.^29^ used experts to delineate esophageal lesions as input for an algorithm. An overlap of at least four delineations was considered to be an area of high suspicion whereas one delineation was considered to be low suspicion.^29^ These data were then used to assess the performance of the algorithm.^29^ Per image analysis showed accuracy of 91.7%, sensitivity of 95% and specificity of 85%.^29^ The AI model had a localization score of 95% for low suspicion areas and 92.5% for high suspicion areas.^29^ The model was effectively used in real-time endoscopic detection of early Barrett's neoplasia and was useful as a guide to identify the best sites to target biopsies.^29^ De Groof *et al*.^30^ then went on to develop a hybrid ResNet-U Net model classifying images as neoplasms or non-dysplastic BE based on five independent datasets. This CAD outperformed all of the non-expert endoscopists in accuracy of detecting lesions and identified the optimal site for biopsy in 97% and 92% of cases in a dataset of 40 images delineated by three experts and 40 images delineated by six experts.^30^ de Groof *et al*.^30^ also used an AI system in real-time endoscopy obtaining multiple image-based CAD predictions at 2 cm intervals. A per-image analysis showed accuracy, sensitivity and specificity of 84%, 76% and 86%, respectively. The system was able to correctly identify 9 out of 10 neoplastic lesions when combining 3 consecutive still images.^30^

Hashimoto *et al*.^39^ also developed a CNN to identify dysplasia in BE. Two expert endoscopists annotated neoplastic images, this was used as the ground truth for the training of the algorithm.^39^ Per image AI identification of dysplasia versus non-dysplasia had accuracy, sensitivity and specificity of 95.4%, 96.4% and 94.2%, respectively, with 24 of 26 patients correctly diagnosed with dysplasia.^39^ The value of this system was its successful use in real-time endoscopy.^39^

More recently, Abdelrahim *et al*.^40^ utilized CNN technology in a multicenter study using real-time endoscopic videos to detect BE and had accuracy of 92%. This system was superior to non-expert endoscopists, especially in identifying flat lesions, however the negative predictive value for low-grade dysplasia, high-grade dysplasia and adenocarcinoma was 95.1%.^40^ This is lower than the 98% per-patient NPV recommendation from The Preservation and Incorporation of Valuable Endoscopic Innovations standards of the American Society for Gastrointestinal Endoscopy (ASGE). However, this value may be increased if the AI model was applied to a population with a lower prevalence of neoplasia and therefore may still be beneficial in screening for BE.^40^

### Further applications of AI in advanced endoscopic imaging techniques

#### High-resolution micro-endoscopy

High-resolution micro-endoscopy (HRME) is a low-cost technique developed to image the epithelium at a cellular level, competing with chromoendoscopy as a screening tool for ESCC.^41^ Shin *et al*.^10^ used this technique to identify neoplastic and non-neoplastic squamous esophageal mucosa. Prior to HRME, a nuclear stain was applied to the mucosa. A two-class linear discriminant algorithm was then developed to identify pathological lesions. The AI system had sensitivity and specificity of 84% and 95% in the validation set.^10^ Quang *et al*. also used HRME in 2016 to identify ESCC. They had comparable results to Shin *et al*. with sensitivity of 95% and specificity of 91%.^10^^,^^42^ The benefit of this technique is the potential low-cost and accurate diagnosis of early ESCC without the need for multiple biopsies or skills to interpret HRME images.^10^

HRME has also been used in patients with BE, providing images with a resolution approaching that of conventional histopathology. Shin *et al*.^43^ had a per-biopsy analysis that resulted in sensitivity of 88% and specificity of 85%.

### Optical coherence tomography

Optical coherence tomography (OCT) is a technique that provides a high-resolution image of esophageal mucosa by the addition of a fiber-optic catheter probe inserted through a standard endoscope.^33^ Previously, the accuracy of endoscopists using this technique has been insufficient to make clinical decisions; however, a CAD system was developed to possibly improve the usefulness of this method in diagnosing dysplasia in BE.^33^ The results of the study showed that the CAD system had accuracy of 83%, sensitivity of 82% and specificity of 74%.^33^ With further refinements of the system, there is potential for this AI system to be a valuable tool in the future.

## DISCUSSION

The early diagnosis of esophageal cancer has a notable effect upon available curative treatment options and thus overall prognosis. Endoscopy remains the gold standard for diagnosing esophageal cancer by providing both a visual and histological diagnosis. Endoscopy services are often under a lot of strain due to the volume of work and the expected level of expertise. Early esophageal cancer often has subtle mucosal features which require expert endoscopy skills and interpretation. In addition, BE surveillance can itself be time-consuming due to the need for multiple sequential biopsies. Due to these challenges, AI has increasingly been incorporated into the endoscopic diagnosis of esophageal cancer. This systematic review and meta-analysis summarizes the evidence of 45 studies analyzing the use of AI as an adjunct to diagnosing esophageal malignancy. For ESCC, 24 studies were included in this review, of which 14 studies contributed to the meta-analysis, with pooled sensitivity and specificity of 91.2% and 80%, respectively. For EAC, 15 studies were included in this review, of which 9 studies contributed to the meta-analysis with pooled sensitivity of 93.1% and specificity of 86.9%. Sixteen out of 24 ESCC studies and 10 out of 15 EAC studies had independent or external validation datasets to assess their AI algorithms.^11^^,^^12^^,^^14^^,^^16–23^^,^^27^^,^^28^^,^^30^^,^^31^^,^^35^^,^^36^^,^^38–40^^,^^42–46^ Validation dataset size ranged from 52 to 2123 cases for ESCC and 9 to 199 cases for EAC.^11^^,^^35^^,^^38^^,^^44^

Overall, there is good evidence to support the use of AI in the endoscopic diagnosis of esophageal cancers. The application of the AI algorithms showed promising results in both WLI and NBI as well as other imaging modalities such as HRME and OCT.

### Comparison with endoscopists

Twenty-nine studies from the included qualitative and quantitative literature included a comparison of AI efficacy with endoscopists. Overwhelmingly, the review largely showed comparable or superior outcomes for AI algorithms compared with endoscopists, with the greatest benefit of AI use being seen as an adjunct to non-expert endoscopists. Shiroma *et al*.^17^ showed that the sensitivities of 13 out of 18 endoscopists improved by a median of 10% using AI real-time assistance during endoscopy.

### Speed of interpretation

A noteworthy finding is that AI outperformed endoscopists in terms of the speed of image interpretation, with Tang *et al*.^21^ showing that their AI system only required 15 milliseconds per image to diagnose an esophageal lesion and additionally outperforming endoscopists in terms of accuracy, sensitivity and specificity. In terms of depth of invasion, Nakagawa *et al*. and Tokai *et al*. demonstrated that their AI models were not only superior to endoscopists but were importantly faster at image interpretation (29 seconds and 10 seconds, respectively, compared with 115 minutes).^3^^,^^12^ The importance of the speed of interpretation will allow for more direct clinical integration, to give potential feedback to an endoscopist in real-time, and advise them on the notable suspicious areas for biopsy.

### Limitations

Errors in the results obtained by AI technology are often related to shadows or anatomical structures such as the esophagogastric junction, the left main bronchus or vertebra. Benign lesions such as resection scars and atrophy may also cause false positive results. False negative interpretation may result from technical errors where the lesion is not fully visualized or from background esophageal inflammation.^34^ With improved image capturing and larger training datasets, more robust AI models can be developed to potentially overcome these errors.

The majority of the included studies used still images, or a combination of still image and video analysis. Still images do not emulate real-life endoscopy and as such videos are more accurate to assess the true capabilities of an AI system. In addition, most of the images used in these studies were obtained by experts and test samples were supplemented with subtle lesions, potentially accounting for significant selection bias. In practice, poorer quality sample images are produced, therefore the current study outcomes limit the use of AI’s ability to be accurately applied to community practice endoscopy.

Another limitation identified in this systematic review and meta-analysis is that only English language articles were included. Given that much of the literature is from Asian centers, perhaps casting a wider net in terms of languages may have captured a broader range of studies. Furthermore, given the geographical variation in types of esophageal cancer, with the Far East having more cases of ESCC, international implementation of the findings of this review may not be entirely applicable. In addition, many of the articles, especially for adenocarcinoma, have data contributions by the same group in different years, and therefore, their findings may be less generalizable to other centers.

It must be emphasized that almost every study included in the meta-analysis had a unique AI algorithm, a variable study design, independent datasets used for training and validation processes and different endoscope models used to obtain images (Olympus, Fujifilm and Pentax), this heterogeneity limits scientific value when pooling these studies in a meta-analysis. Overall, there was evidence of moderate heterogeneity with i2 values of 67.3 and 72.5 for Figures 3 and 5, respectively.

## CONCLUSION

AI shows great promise in improving diagnostic capabilities for esophageal malignancy, in particular, early diagnosis of cancer. The value of accurate detection of early stage malignancy offers patients the potential for curative treatment endoscopically, with survival outcomes similar to surgery, and a substantially improved Health Related Quality of Life through an organ preservation strategy. This article highlights many developments in AI technology with the potential to revolutionize esophageal malignancy diagnosis and management, however, higher quality of evidence is required first before such technology can be implemented into standard patient care.

## Notes

Financial support: None.
